# EPS-Producing *Lactobacillus* *plantarum* *MC5* as a Compound Starter Improves Rheology, Texture, and Antioxidant Activity of Yogurt during Storage

**DOI:** 10.3390/foods11111660

**Published:** 2022-06-05

**Authors:** Xuefang Zhao, Qi Liang

**Affiliations:** Functional Dairy Products Engineering Laboratory of Gansu Province, College of Food Science and Engineering, Gansu Agricultural University, Anning District, Lanzhou 730070, China; zhaoxuefang2022@hotmail.com

**Keywords:** *Lactobacillus plantarum MC5*, exopolysaccharide (EPS), yogurt, rheological properties, antioxidant activity

## Abstract

This study evaluated the effects of probiotic *Lactobacillus plantarum* *MC5* on the quality, antioxidant activity, and storage stability of yogurt, to determine its possible application as a starter in milk fermentation. Four groups of yogurt were made with different proportions of probiotic *L. plantarum* *MC5* and commercial starters. The yogurt samples’ rheological properties, texture properties, antioxidant activity, storage stability, and exopolysaccharides (EPS) content during storage were determined. The results showed that 2:1 and 1:1 yogurt samples (supplemented with *L. plantarum* *MC5*) attained the highest EPS content (982.42 mg/L and 751.71 mg/L) during storage. The apparent viscosity, consistency, cohesiveness, and water holding capacity (WHC) of yogurt samples supplemented with *L. plantarum* *MC5* were significantly higher than those of the control group (*p* < 0.05). Further evaluation of antioxidant activity revealed that yogurt samples containing *MC5* starter significantly increased in DPPH, ABTS, OH, and ferric iron-reducing power. The study also found that adding *MC5* can promote the growth of *Streptococcus thermophilus*. Therefore, yogurt containing *L. plantarum* *MC5* had favorable rheological properties, texture, and health effects. The probiotic *MC5* usage in milk fermentation showed adequate potential for industrial application.

## 1. Introduction

Fermentation is an old process of food preparation in the world whereby the growth and activity of microorganisms are used to preserve foods [1]. Yogurt is widely manufactured throughout the world, and approximately 400 generic names exist for traditional and commercial products [2]. According to the “2020 Yogurt Market Research Report” released by QY Research, the global yogurt market has reached USD 70 billion in 2019 [3]. Under the current background of great health, probiotic fermented dairy products are the first choice of consumers due to their health benefits, particular sensory properties, and extended shelf life [4]. These beneficial effects are closely related to probiotics and their metabolites. Exopolysaccharide (EPS) is a polymer with a high molecular mass metabolized in situ by specific lactic acid bacteria (such as *Streptococcus*, *Lactobacillus*, *Lactococcus*, and *Leuconostoc*). These EPS can covalently bind to the cell surface of lactic acid bacteria to form capsules [5], either loosely attached to its surface, or fully secreted into the surrounding environment during the growth of the strains. Among the wide variety of EPS-producing microorganisms, lactic acid bacteria (LAB) are generally considered safe due to their long history of use in human consumption [6,7]. It has been reported that its application to milk fermentation can increase viscosity [8]. In addition to maintaining bacterial homeostasis and promoting bacterial survival, research on EPS-producing LAB has focused on the health-promoting effects of EPS, such as immunoregulatory activity [9], antioxidant [10], and anti-tumor [11].

Currently, traditional fermented milk remains a natural, biodiverse resource bank for the search for “new” strains with technical and functional properties [12]. *L. plantarum MC5* is a strain with various ideal functions isolated from traditionally fermented yak yogurt in domestic Tibetan areas by Zhu et al. [13]. These include inhibiting pathogenic bacteria, producing EPS, anti-oxidation, and tolerating simulated gastrointestinal fluid. Among the various functional properties of probiotic *MC5*, EPS-producing ability is one of the most important properties. Wang et al. [14] found that EPS produced by *L. plantarum* YW11, isolated from Tibetan kefir, had higher viscosity and water retention capacity when skim milk was treated with lower temperature, acidic pH, and shear positive contact behavior induction. Wang [15] reported that EPS isolated from *Leuconostoc mesenterica* XR1 can be used as a natural organic additive to replace chemical additives in dairy products. In addition, the EPS-producing LAB can also improve the sensory properties of fermented milk [16]. Therefore, screening new EPS-producing strains and renewing old strains’ resources is very valuable for maintaining biodiversity.

*Lactobacillus plantarum* has been reported as a producer of EPS with various properties and activities essential for commercialization by the food, cosmetic, or pharmaceutical industries [9,17]. On the one hand, EPS-LAB application in yogurt production is expected to increase due to consumers’ high demand for fermented foods with minimal or no chemical additives, smooth texture, and good sensory properties [18]. Again, most reports on EPS-producing LAB so far have focused on the functional characteristics of EPS. However, the influence of EPS-producing LAB as a compound starter on milk fermentation and the compound effect between the EPS-producing probiotics and commercial starters (*S. thermophilus* and *Lb. delbrueckii* subsp. *bulgaricus*) has not been well studied. Therefore, this study aims to investigate the effect of *MC5* as a compound starter on the texture properties (firmness, consistency, and cohesiveness), rheological properties (apparent viscosity, elasticity, and stickiness), storage stability, and antioxidant activity of yogurt during storage. Thus, to determine the fermentation characteristics of *MC5* and the interaction between EPS-*MC5* and commercial starters in yogurt samples. This study provides information on the EPS-producing strains with the best technical capabilities (fermentation characteristics and health benefits) to enhance their applications in the dairy industry.

## 2. Materials and Methods

### 2.1. Materials and Reagents

Cow milk was collected from the dairy farm of Gansu Agricultural University. The contents of fat, solid not fat (SNF), protein, lactose, ash content, and total solids in Holstein milk were measured by a milk composition analyzer MCCWV1 (Hangzhou Melisco Technology Co., Ltd., Hangzhou, China). The composition of Holstein milk is shown in Table 1. *L. plantarum* *MC5* was isolated from traditional fermented yak yogurt in Tibet [13], Gansu province. Strains *MC5* were inoculated into skim milk-glycerol tubes and stored at −80 °C; yogurt starter (including *Streptococcus thermophilus* and *Lactobacillus delbrueckii* subsp. *bulgaricus*, low viscosity type) was obtained from Kunshan Baishengyou Biotechnology Company, Ltd., Kunshan, China).

MRS broth [19]: peptone (10 g/L), beef extracts (10 g/L), yeast extract (5 g/L), glucose (25 g/L), lactose (25 g/L), Tween 80 (1 mL/L), K_2_HPO_4_ (2 g/L), sodium acetate (5 g/L), diammonium hydrogen citrate (2 g/L), MgSO_4_ (0.2 g/L) and MnSO_4_ (0.08 g/L). It was sterilized at 121 °C for 20 min.

M17 broth [19]: fish peptone (5 g/L), peptone (2.5 g/L), casein peptone (2.5 g/L), beef extracts (5 g/L), yeast extract (5 g/L), lactose (3 g/L), MgSO_4_ (0.25 g/L), KH_2_PO_4_ (5 g/L) and sodium ascorbate (0.5 g/L). It was sterilized at 121 °C for 20 min.

Skim milk: skim milk powder (100 g/L), yeast extract powder (1 g/L), distilled water (100 mL). It was sterilized at 115 °C for 15 min.

### 2.2. Culture and Incubation of LAB

The *L. plantarum MC5* were inoculated 4% (*w*/*v*) in sterilized and cooled skim milk and cultured at 140 rpm at 37 ◦C for 24 h. Then they were inoculated 4% (*w*/*v*) in MRS broth (in Section 2.1) for 2 generations (37 °C, 24 h) for subsequent experiments.

Commercial starters (*Streptococcus thermophilus* and *Lactobacillus delbrueckii* subsp. *bulgaricus*) were inoculated 4% (*w*/*v*) in sterilized and cooled skim milk and cultured at 42 °C for 12 h, respectively. Then they were inoculated 4% (*w*/*v*) in M17 broth (in Section 2.1) for 2 generations (42 °C, 12 h) for subsequent experiments.

### 2.3. Production of Coagulated Yogurt

Starter cultures: skim milk (14% *w*/*v*) was sterilized at 115 °C for 15 min. They were cooled and inoculated with *L. plantarum MC5* and commercial starters (*Streptococcus thermophilus* and *Lactobacillus delbrueckii* subsp. *bulgaricus*), the inoculum amounts of *L. plantarum MC5* and commercial starters are shown in Table 2. Then they were cultured at 42 °C to pH 4.6.

**Figure 1:**
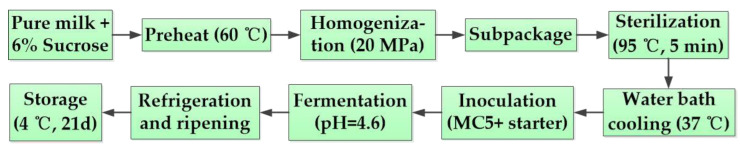
The production process of yogurt.

The production of the coagulated yogurt method by Heena et al. [20] was followed. Six (6)% sucrose was added to the cow milk, the mixture was preheated at 60 °C for 15 min, followed by cooling to 37–40 °C. It was divided into glass yogurt bottles, thermally treated at 95 °C for 5 min and cooled to 37 °C (desirable temperature for culture addition). Then, starter cultures were inoculated 3% (*w*/*v*) into the cow milk (starter cultures were mixed very slowly in the milk with a ladle). The filled cups were then incubated at 42 °C till pH reached around 4.6 ± 0.02, at which point the fermentation time was 5.5–6 h. Finally, yogurt samples were stored at refrigeration temperature (4 ± 1 °C) for 21 days (Figure 1). Total yogurt samples were 48. Analyses were performed on the yogurt samples after 1, 7, 14, and 21 days of storage. The experiment was repeated in triplicate.

### 2.4. Determination of EPS in Yogurt

The EPS content was determined as follows: 10 mL of yogurt simple was heated in a 100 °C water bath for 15 min and then centrifuged (8000 r/min, 4 °C for 20 min). The supernatant (5 mL) was mixed with 20% (*w*/*v*) trichloroacetic acid (TCA), allowed to stand at 4 °C for 24 h, and then centrifuged again. The supernatant was collected, mixed with 95% alcohol (3:1 *v*/*v*), and allowed to stand again at 4 °C for 24 h. The mixture was centrifuged, the pellet was suspended in deionized water and dialyzed at 4 °C for 2 days using dialysis bags (molecular weights of 8 kDa-14 kDa). Afterward, the dialysate was concentrated and vacuum freeze-dried. The EPS concentration in the suspension after dialysis was quantified using the phenol-sulphuric method of Charchoghlyan et al. [21], and is expressed as glucose equivalent with glucose as the standard [22].

### 2.5. Analysis of Rheological Properties of Yogurt

#### 2.5.1. Apparent Viscosity

Apparent viscosity was determined for all samples using an MCR301 Rheometer (Anton Paar, Glaz, Austria). The yogurt sample was linearly sheared at a constant temperature of 25 °C, the shear rate was 0.1 to 200/s, the measurement time was 1 min, and the change of the apparent viscosity of the samples with the shear rate was detected [23].

#### 2.5.2. Shear Scan of Yogurt

The yield stress was determined for all samples using the MCR301 Rheometer (Anton Paar, Austria). Yield stress implied that stress had to be applied to the viscoelastic material until it began to flow. Stress–strain rate curves of the upward/downward strain rate sweep test were fitted using the Herschel–Bulkley model [21], which is used to elucidate the yielding behavior of viscoelastic materials and is calculated using:

σ = σ_o_ + K·γ^n^,

σ: is shear stress as a function of shear rate;

σ_o_: is yield stress;

K: is the consistency index;

n: is the flow behavior index.

#### 2.5.3. Determination of Thixotropic Properties (TP) and Viscoelasticity of Yogurt

The TP of yogurt samples was measured by using MCR301 Rheometer in the range of 0–200 rad/s. The shear stress of yogurt samples was measured under the condition of 5% strain force and frequency of 0.1–100 Hz [15].

### 2.6. Analysis of Texture Properties of Yogurt

Texture Profile Analysis (TPA) was performed on all samples using Texture Analyzer TA.XT Express (Stable Micro Systems, Guildford, UK). Yogurt samples stored at 4 °C were evaluated for parameters such as firmness, consistency, and cohesiveness using an extrusion unit [24]. All assays were performed in triplicate.

### 2.7. In Vitro Determination of Antioxidant Activity of Yogurt

The yogurt samples and 95% ethanol were thoroughly mixed at a mass ratio of 1: 9 to prepare test samples.

#### 2.7.1. Determination of DPPH Radical Scavenging Activity (RSA) in Yogurt

The RSA of DPPH method of Aguilar et al. [25] was followed. Sample (1.0 mL) was mixed with DPPH-ethanol solution (2.0 mL, 0.2 mmol/L) evenly, placed in the dark for 30 min, centrifuged (8000 r/min, 10 min) and the absorbance of the supernatant was measured at 517 nm (*A_j_*). Blank was measured using ultrapure water (1 mL) and anhydrous ethanol (2 mL). The RSA of DPPH was calculated using:

$$\mathrm{RSA}\;\mathrm{of}\;\mathrm{DPPH}\;(\%)=(1-\frac{A_{j}-A_{i}}{A_{o}})\;\times \;100\%,$$

*A_j_*: Absorbance of ultrapure water (1 mL) + EPS solution (1 mL);

*A_i_*: Absorbance of DPPH-95% ethanol (1 mL) + EPS solution (1 mL);

*A_o_*: Absorbance of DPPH-95% ethanol + ultrapure water (1 mL).

#### 2.7.2. Determination of ABTS Radical Scavenging Activity (RSA) in Yogurt

ABTS solution was prepared by mixing equal volumes of ABTS (7 mmol/L) and potassium persulfate solutions (2.45 mmol/L), and the mixture was placed in the dark for 16 h. Before the assay, the ABTS radical solution was diluted with PBS (0.2 mol/L, pH 7.4) and its absorbance was standardized to 0.70 ± 0.02 at the wavelength of 734 nm. Sample solution (0.4 mL) was added to 3 mL of the diluted ABTS solution and allowed to stand in the dark for 10 min. The absorbance of the sample solution was measured at a wavelength of 734 nm [26]. The formula for calculating the RSA of ABTS is as follows:

$$\mathrm{RSA}\;\mathrm{of}\;\mathrm{ABTS}\;(\%)=(1-\frac{A_{s}-A_{c}}{A_{o}})\;\times \;100\%,$$

*A_s_*: Absorbance of the sample + ABTS solution;

*A_c_*: Absorbance of ABTS solution + ultrapure water;

*A_o_*: Absorbance of sample + ultrapure water.

#### 2.7.3. Determination of Hydroxyl (OH) Radical Scavenging Activity (RSA) in Yogurt

The Fenton method reported by Du et al. [27] was followed to determine the RSA of OH. To do this, 0.5 mL of 2.5 mmol/L phenanthroline solution, 0.5 mL of 2.5 mmol/L FeSO_4_ solution and 0.5 mL of 20 mmol/L H_2_O_2_ solution were sequentially added in 1 mL of 0.02 mol/L PBS (pH 7.4) solution and thoroughly mixed before addition of 0.5 mL of sample. The absorbance of the sample was measured at 536 nm after heating it in the water bath at 37 ℃ for 1 h. The formula for calculating the RSA of OH is as follows:

$$\mathrm{RSA}\;\mathrm{of}\;\mathrm{OH}\;(\%)=(1-\frac{A_{s}-A_{c}}{A_{b}})\;\times \;100\%,$$

*A_s_*: Absorbance of sample + H_2_O_2_;

*A_c_*: Absorbance of ultrapure water + H_2_O_2_;

*A_b_*: Absorbance of ultrapure water.

#### 2.7.4. Determination of Ferric Iron-Reducing Power (FRP) in Yogurt

To 1 mL of the sample solution, 1 mL of phosphate buffer (pH 6.6, 0.2 mol/L) and 1 mL of potassium ferricyanide solution (1%, *w*/*v*) were added and mixed thoroughly. The mixture was kept at 50 °C for 20 min, 1 mL of TCA (10%, *w*/*v*) was added, and the mixture was centrifuged (3000 r/min, 10 min). To 2.5 mL of supernatant, 2.5 mL of deionized water, and 0.5 mL of ferric iron chloride solution (0.1%, *w*/*v*) were added and mixed. After standing for 10 min, the absorbance was measured at 700 nm [28]. The FRP is represented by the absorbance value, and the greater the absorbance value, the stronger the reducing power. The formula for calculating the RSA of OH is as follows:

$${OD}_{700}=A_{1}\;-\;A_{0}\text{,}$$

*A*_1_: Absorbance of sample;

*A*_0_: Absorbance of the blank.

### 2.8. Microbiological Analysis in Yogurt

Yogurt samples were subjected to microbiological analysis on days 1, 7, 14, and 21. All microorganisms previously put into the milk for fermentation samples were enumerated by using different media and methods described below, yogurt (1 mL) was mixed with 9 mL of normal saline (0.85%), homogenized, and diluted at a 10-fold gradient. From three chosen dilutions, 10^−6^, 10^−7^, and 10^−8^, 0.1 mL of each was taken and evenly spread on the plate culture, and the total number of colonies was counted [29].

#### 2.8.1. Streptococcus Thermophilus

M17 agar medium was used to enumerate *S. thermophilus*. Using the medium at pH 6.9 ± 0.20, the inoculated plates were incubated at 42 °C for 48 h.

#### 2.8.2. *L. delbrueckii* subsp. *bulgaricus*

*L. delbrueckii* subsp. *bulgaricus* was cultured in an MRS agar medium (adjusted to pH 5.2 with glacial acetic acid) at 42 °C for 48 h.

#### 2.8.3. *Lactobacillus plantarum* *MC5*

For samples supplemented with *L. plantarum* *MC5* and commercial starters, the corresponding number of *S. thermophilus* and *Lb. delbrueckii* subsp. *bulgaricus* was subtracted. Then *L. plantarum MC5* was cultured in an MRS agar medium supplemented with 0.01% (mass fraction) *Staphylococcus aureus* at 37 °C for 48 h. They were all cultured under anaerobic conditions.

### 2.9. Analysis of pH, Titratable Acidity (TA), and Water Holding Capacity (WHC) of Yogurt

The pH value of samples was measured at room temperature with a PHS-3C pH-Meter and a combined glass electrode (Shanghai INESA Scientific Instrument Co., Ltd., Shanghai, China).

The TA was determined by the Chinese National Food Safety Standard-Determination of Food Acidity (GB 5009.239-2016) [30] using phenolphthalein as an indicator using.

The WHC was determined using Paulina [31] with slight modifications. Yogurt samples (20.0 g) were accurately weighed and centrifuged at 5000 r/min for 10 min. The supernatant was discarded, the residue was weighed, and WHC was calculated according to the formula:

$$\mathrm{WHC}\;(\%)=\frac{W_{1}-W_{2}}{W_{1}}\;\times \;100\%,$$

*W*_1_: weight of samples before centrifugation/g;

*W*_2_: weight of samples after centrifugation/g.

### 2.10. Statistical Analysis

All tests were performed in triplicate. Each sample was analyzed in triplicate. The total number of yogurt samples was 48 (4 yogurt types × 3 repeated measurements × 4 sampling time). Data were analyzed using the mixed linear model of SPSS 22.0 (Statistical Package for the Social Sciences, Chicago, IL, USA). The statistical models included fixed effects treatment (*MC5* inclusion level), sampling days (time), interaction (Trat × T), and random effect repeated measures (sample ID). ANOVA tests were used to determine significant differences between treatments with a significance level of *p* < 0.05 by the SPSS 22.0 package program The figures were drawn using the Origin 8.0 software (Statistical Package for the Social Sciences, Hampton, MA, USA). The PCA results are presented both as a variables map (correlation between studied variables) and as an individual map (similarity between yogurt samples).

## 3. Results and Discussion

### 3.1. Content of EPS in Yogurt

**Figure 2:**
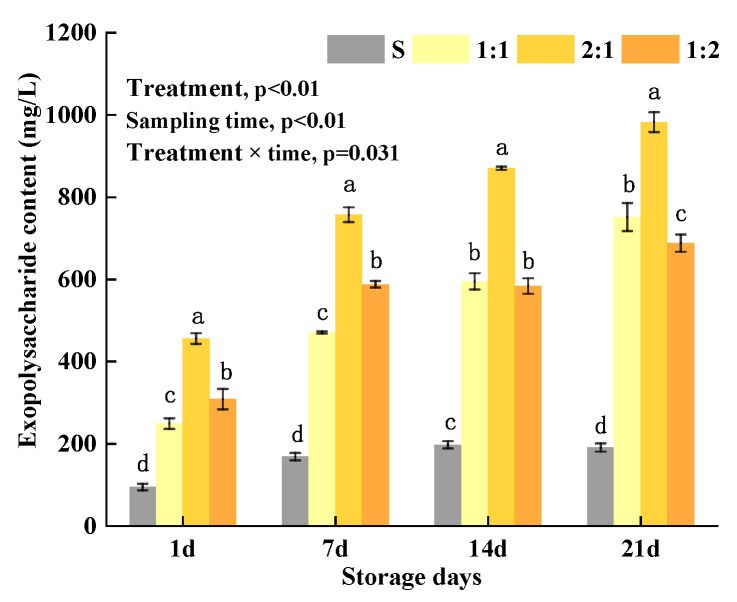
Content of EPS in yogurt with different proportions of *MC5* added during storage. The control group S only has added commercial starters, the 1:1, 2:1, and 1:2 groups denote that the proportion of adding *MC5* and commercial starters is in a ratio of 1:1, 2:1, and 1:2, respectively. Error bars represent the standard errors (se) of the model-fitted mean value (*n* = 3). “a, b, c, d” indicate significant differences within yogurt groups during storage at *p* < 0.05.

EPS produced by LAB can change the rheological properties, texture properties, and taste of food [32]. The continual protein rearrangements in yogurt during storage and the greater number of protein–EPS–protein contacts established result in a more solid-like gel; thus, the presence of EPS channels in the serum confer a more polymer-like rheological behavior [33]. *Dahi* containing EPS-producing LAB had the maximum acceptability and highest sensory scores during storage [34]. Therefore, they are used in the food industry as tackifiers, stabilizers, emulsifiers, or gelling agents [35]. The results showed that EPS production in *MC5* and commercial starter samples was significantly higher than those of the control S samples during storage (Figure 2) (*p* < 0.05). After 21 storage days, the EPS production in 1:1, 2:1, and 1:2 samples increased by 2.0, 1.2, 1.3 times more than those of the S samples, which showed that adding *L. plantarum* *MC5* significantly contributed to the EPS production to yogurt samples. The EPS production in the four yogurt samples gradually increased during the storage: control S increased by 50.44%, while 1:1, 2:1 and 1:2 samples increased by 66.88%, 53.61%, and 55.15%, respectively. The highest EPS production observed were those of 1:1 and 2:1 samples, which were 751.71 mg/L and 982.42 mg/L. These increments were significantly higher than those of the S and 1:2 samples (*p* < 0.05), and this could be attributed to the synergy and antagonism interactions between the LAB. During the storage period, the EPS production in the 2:1 sample was always significantly higher than those of the 1:1 and 1:2 samples (*p* < 0.05) indicating that the 2:1 proportion of *L. plantarum MC5* formulation was most effective in increasing the EPS production. Heena et al. [20] showed that protein and amino-acid metabolism enhanced over 0–14 days of goat milk yogurt storage, while fatty acid biosynthesis metabolism predominated during 14–28 days of storage. They also reported that upregulated metabolites included amino acids and peptides during 0–14 days, while saccharides and carboxylic acids were observed during 14–28 days. Furthermore, the *Lactobacillus delbreuckii* acted on cow milk proteins (mainly casein) and hydrolyzed these to free amino acids and peptides, while *S. thermophilus* helped in the formation of different metabolites such as pyruvic acid, formic acid, fatty acids, etc., for the growth of *Lactobacillus spp*. and production of EPS [36].

It was also observed that the increment of EPS in the 2:1 group was higher than in the 1:1 group. The 1:2 group increased rapidly in the first 7 days and then stabilized. This may be due to the high concentration of nutrients available for yogurt LAB growth in the first 7 storage days, while those substances were used-up after the 14 storage days. The interaction between the addition ratio of *MC5* and the storage time of yogurt samples had a significant effect on content of EPS (*p* < 0.05). Schmidt [20] reported that the highest EPS content of yogurt fermented with a combination of *S. thermophilus* and *L. delbrueckii* was 250 mg/kg after storage for 21 days. These results were lower than that of this current study and could most likely be due to differences in starters culture and, partly due to the milk treatment differences. In addition, Pachekrepapo has also shown that EPS produced by different species or different strains of the same species may have different yields, repeating unit structures, and molar masses during fermentation [37]. This study supports a previous study by Zannini [35] as the authors reported that EPS-producing LAB as a compound starter significantly increased EPS yield during storage.

### 3.2. Rheological Properties of Yogurt

#### 3.2.1. Apparent Viscosity

**Figure 3:**
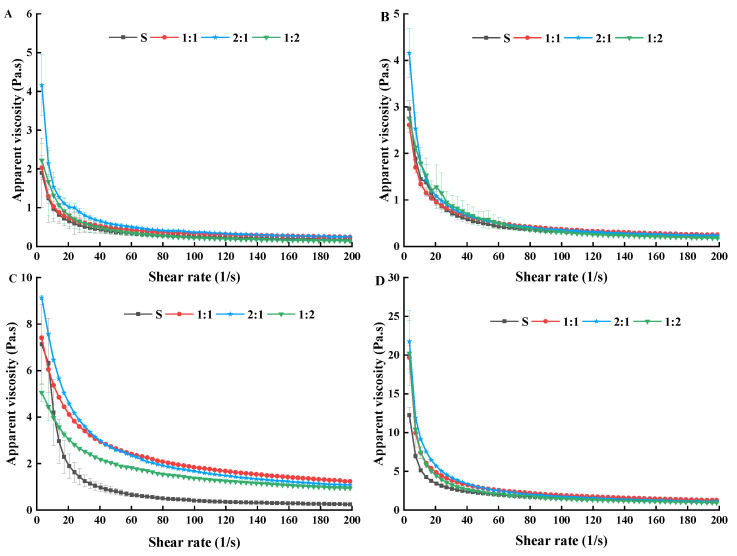
Changes in apparent viscosity of yogurt supplemented with different proportions of *MC5* during storage. (**A**–**D**) Apparent viscosity of the four groups of yogurt milk on the 1st, 7th, 14th, and 21st days in storage, respectively. Error bars represent the standard errors (se) of the model-fitted mean value (*n* = 3). “a, b, c, d” indicate significant differences within yogurt groups during storage at *p* < 0.05.

To study the influence of the shear rate on yogurt gel properties, the shear stability of the four groups of yogurt was assessed. As shown in Figure 3, under the shear rate of 0~200/s, similar trends in apparent viscosities were observed in all the yogurt samples. The apparent viscosity of each group of samples was initially high and then decreased with increasing shear rate and time until it finally stabilized, indicating a correlation between the shear-thinning phenomenon, apparent viscosity, and time. The apparent viscosity in the 1:1, 2:1, and 1:2 groups was significantly higher than that of the S sample during the storage (*p* < 0.05), the highest apparent viscosity attained in the 1:1, 2:1, and 1:2 samples was 19.69 Pa.s, 21.76 Pa.s, and 20.23 Pa.s, respectively (after 21st storage day). In addition, when the shear rate was increased from 0 to 60/s, the apparent viscosity of control S decreased at a faster rate than the decreasing rates of 1:1, 2:1, and 1:2 samples. This may be due to the external force disruption of the balanced structure formed by unknown particles in yogurt suspension [38]. When the shear rate was extended higher than 60/s, the apparent viscosity of the four groups of samples decreased slowly and gradually stabilized. This implied that the four groups of samples showed characteristics of shear dilution, as well as constant and ideal Newtonian fluid behavior [39]. This characteristic of fermented strains has important applications for dairy processing.

During storage, the apparent viscosity of S, 1:1, 2:1 and 1:2 samples significantly increased from 1.90 Pa.s to 12.24 Pa.s, 2.03 Pa.s to 19.69 Pa.s, 4.16 Pa.s to 21.76 Pa.s, and 2.22 Pa.s to 20.23 Pa.s during 21 storage days, respectively. Even though the apparent viscosity of control S also increased, its increment was slower than those of the 1:1, 2:1, and 1:2 samples. In addition, the apparent viscosity of 1:1, 2:1, and 1:2 samples increased slowly before the 14 days of storage but increased rapidly after the 14 days of storage. Therefore, the results showed that the probiotic *MC5* has stable apparent viscosity and tissue structure in yogurt samples. In addition, the apparent viscosity of yogurt samples was influenced by the power and number of bonds between casein micelles of yogurt, as well as their texture and spatial sharing [40]. Comparing the production of EPS in yogurt revealed that the higher apparent viscosity was caused by the accumulation of a large number of EPS in the situ metabolism of *MC5* (especially in the last 7 days), as well as the formation of a protective film around *MC5*. In addition, Doleyres et al. [41] reported that in situ EPS production by the LAB could be a better approach than adding EPS as bioingredient to improve rheological characteristics of yogurt, which could also explain the better rheological properties observed in our study. At the same time, the results of Chaudhury [34] showed that the sensory quality of fermented milk produced by EPS-producing strains were the best. This suggested that EPS macromolecules added to milk before fermentation can interfere with acid coagulation and gel network formation differently than EPS produced in situ during milk acidification. Our research team previously found that the *MC5* had good tolerance (with a survival rate of 88.32% and 69.03% after 3 h in simulated artificial gastrointestinal fluids). This may be because the EPS produced by *MC5* formed a protective film on the surface of the bacteria, so it had a higher survival rate [5].

Furthermore, the highest apparent viscosity in all stirred yogurts was 1.7 Pa.s in Guénard-Lampron’ study [42], which was less than that of this study. Differences in apparent viscosity of yogurt made from EPS-producing strains could be attributed to differences in the number and the molecular characteristics of EPS and their ability to interact with proteins [43]. Kumar [44] showed that the apparent viscosity of dairy products decreased when the speed increased during agitation. However, when the stirring speed slowed down and finally stopped, the apparent viscosity of the dairy products increased accordingly, which was more conducive to the processing and production of coagulated yogurt. In addition, the yogurt samples of this study have a higher apparent viscosity, because they were coagulated yogurts. Piermaria et al. [45] reported that the milk fermented with all the *L. paracasei* strains presented the highest apparent viscosity; nevertheless, the viscoelastic characteristics of the resulting yogurts were different. Production of high molecular weight exopolysaccharides affects the viscosity of yogurt. In summary, yogurt addition of *MC5* obtained a stronger structure between EPS and casein micelles, which could have a successful application in improving the texture of the fermented dairy products.

#### 3.2.2. Shear Scan of Yogurt

Rheological parameters for yogurt samples were measured using the Herschel–Bulkley model to determine flow behavior. The determination coefficient (R^2^) for 1:1, 2:1, 1:2, and the control S samples ranged from 0.990 to 0.999, which was an acceptable fit for the flow curves (Table 3). The yield force is the maximum shear force that a Newtonian fluid needs to be applied to deform. During storage for 21 days, σ_o_ increased significantly for all yogurt samples. The yield forces of yogurt samples in 1:1 and 2:1 groups were as high as 66.90 Pa and 54.79 Pa. The results in this study were higher than those of Charchoghlyan [21], who reported that the yield stress of BHF, NHF, and NLF yogurt were 7.60 Pa, 5.22 Pa, and 3.99 Pa, respectively. The differences may be due to the type and concentrations of starters used. In addition, interaction between the addition ratio of *MC5* and the storage time of yogurt samples had a significant effect on yield forces and K (*p* < 0.01). The consistency index K, and the flow behavior index n was obtained using the Herschel–Bulkley model [41]. The K value of the consistency coefficient of all samples increased with the increase of the viscosity of yogurt samples. The K of the 1:1, 2:1 and 1:2 yogurt samples increased from 2.51 Pa.s^n^ to 18.79 Pa.s^n^, 4.70 Pa.s^n^ to 49.09 Pa.s^n^, and 5.00 Pa.s^n^ to 19.34 Pa.s^n^, respectively. The yogurt samples in this study were all typical pseudoplastic fluids (*n* < 1), the value of the fluid behavior index showed a decreasing trend with the increase of the viscosity of the yogurt samples. This may be due to the increased accumulation of EPS and the increased intramolecular friction of protein gels in yogurt, which increased the overall viscosity of the corresponding sample. The results indicated that the content of EPS in yogurt samples had a significant effect on K and n, and this was consistent with the results in 2.1. This study supports previous studies reporting that different EPS have different effects on the rheological properties of yogurt [37].

#### 3.2.3. Determination of Thixotropic Properties and Viscoelasticity of Yogurt

**Figure 4:**
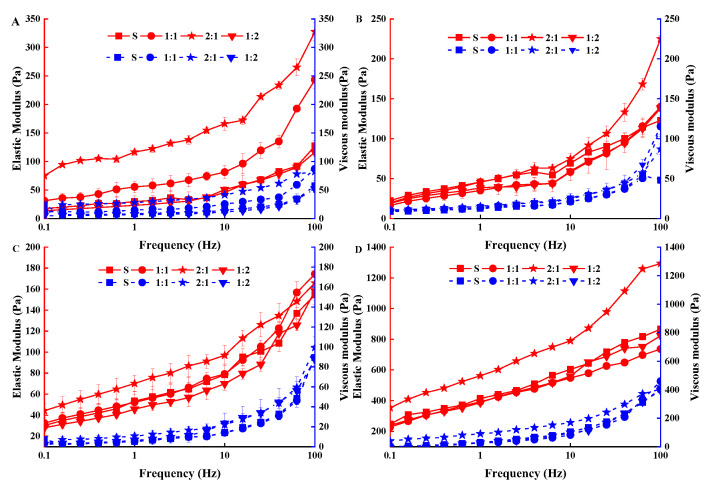
The elastic modulus G’ and the viscous modulus G″ of yogurt were added with different proportions of MC5 during storage. (**A**–**D**) G′ and G″ of the four groups of yogurt on the 1st, 7th, 14th, and 21st days of storage, respectively. Error bars represent the standard errors (se) of the model-fitted mean value (*n* = 3).

In this study, the elastic modulus G’ and viscous modulus G″ of four groups of yogurt samples showed the same variation trend. As the frequency increased from 0.1 Hz to 100 Hz, the elastic moduli G′ and viscous moduli G″ of the four groups of yogurt samples increased gradually. Notably, all samples showed weak viscoelastic gel characteristics because G′ was greater than G″, indicating that the four groups of yogurt had elastic and solid-like characteristics (Figure 4).

During the storage period, G′ of the 2:1 yogurt sample was significantly higher than that of 1:1, 1:2, and control S. In addition, the viscoelastic modulus of the control S slowly increased during the storage, while those of 1:1, 2:1, and 1:2 samples slowly increased before 14 storage days, then rapidly increased after 14 days of storage. These findings suggested that the fermentation performances, as well as viscoelasticity of the yogurts fermented with *MC5* were the best. This may be due to the electrostatic interaction of EPS with casein and whey proteins during the first 14 days of storage, and EPS in yogurt can act as an active filler and increase viscoelastic modulus when interacting with proteins.

The results showed that the high concentration of EPS promoted more incompatibility with the protein network, which might have also led to the decrease in the G’ value of EPS-added yogurts. Zhang et al. [46] also reported that the interaction between EPS and CAS (casein) improved the viscoelasticity of the EPS-YW11/CAS complex with the addition of 1% EPS-YW11. Contrarily to this study, Ibrahim [47] reported that the viscoelasticity of yogurt was related to its gel point. Before gel formation, the presence of EPS in yogurt created macroporosity, leading to a decrease in viscoelastic modulus. After gelation, EPS production did not affect the porosity of the network but might have led to an increase in stiffness. This was due to the difference in the type of LAB, the amount and structure of EPS it produced (such as EPS type, molecular weight, and molecular composition) as well as the process parameters (such as temperature, time, and inoculation amount) yogurt production.

### 3.3. Texture Properties of Yogurt

**Figure 5:**
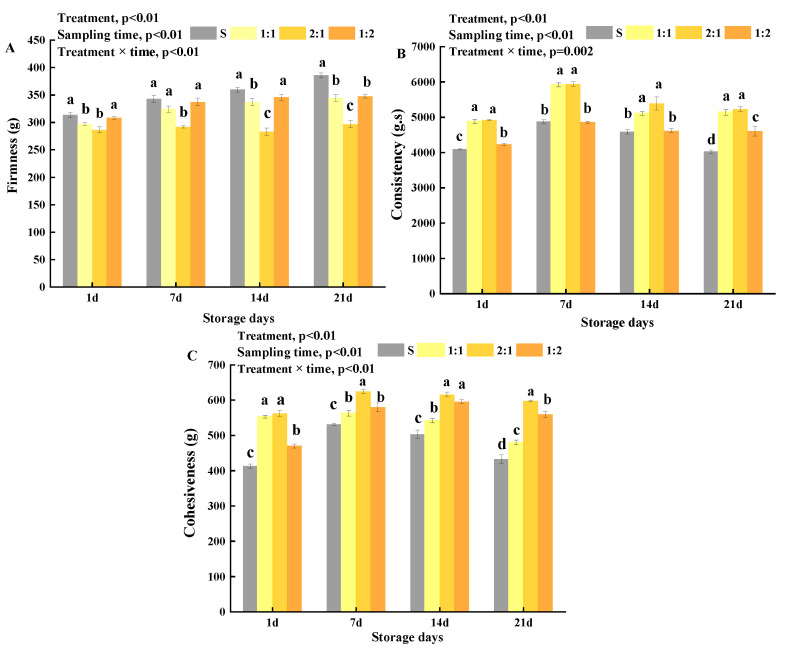
Texture properties of yogurt were added with different proportions of *MC5* during storage. (**A**–**C**) Firmness, consistency, and cohesiveness of yogurt, respectively. Error bars represent the standard errors (se) of the model-fitted mean value (*n* = 3). “a, b, c, d” indicate significant differences within yogurt groups during storage at *p* < 0.05.

In this study, to assess the effect of adding *MC5* on the texture of yogurt samples, the texture of four coagulated yogurt samples during storage was monitored. The parameters measured were firmness, consistency, and cohesiveness. Firmness, a key yogurt quality parameter, is defined as the force required to ensure certain deformation of food ingredients [48]. As shown in Figure 5A, the firmness of the 1:1 and 2:1, and 1:2 groups were lower than that of the control S. The 2:1 sample showed the lowest firmness (296.55 g) after 21 storage days; thus, *MC5* addition significantly reduced the firmness of the yogurt samples. Similar results were reported by Bancalari [49], where it wsa observed that the firmness of yogurts made using EPS-producing starter cultures was generally lower than that of the control yogurt made without EPS-producing starter cultures. Guénard-Lampron [42] reported that the firmness of stirred yogurt was 250–500 N/m^2^ after 1 day of storage. Zhao et al. [50] reported that EPS could affect the textural properties of a yogurt clot by decreasing its firmness. However, the firmness of camel’s yogurt was lower than that of this study, which may be due to the differences in (i) soluble solids contents between camel milk and cow milk, (ii) starters, (iii) gel structures between curdled yogurt and stirred yogurt. The firmness of control S samples increased gradually with the extension of storage time, while those of 1:1, 2:1, and 1:2 samples remained fairly constant during storage. Yogurt samples with high consistency refer to a viscous product with high density. The yogurt samples showed a strong correlation between consistency and viscosity (R = 0.86). After 21 days of storage, the consistency of the 2:1 and 1:1 groups was 5141.05 g·s and 5229.12 g·s, respectively, and these values were significantly higher than those of the S and 1:2 groups (*p* < 0.05, Figure 5B). This observation may be due to the difference in the EPS production and post-acidification of yogurt during storage. Yildiz [24] reported that firmness and consistency were affected by syneresis, pH decrease, and casein hydration increase in yogurts with long storage time. The study also found that the interaction between the addition ratio of *MC5* and the storage time of yogurt samples had a significant effect on firmness, consistency, and cohesiveness of yogurt (*p* < 0.01).

Cohesiveness, a strong binding indicator, affects the structural integrity of yogurt [51]. The cohesiveness in the 1:1, 2:1, and 1:2 yogurt samples was significantly higher than that of the S (*p* < 0.05). The 1:1, 2:1, and 1:2 samples attained maximum levels of 479.96 g, 597.42 g, and 559.56 g at the end of storage, respectively (Figure 5C). This observation was most likely due to the myofibril filaments-like network formed between the fat globules embedded in the protein matrix, and the EPS produced by *MC5* which connected the microbial cells and the yogurt protein matrix, forming a relatively stable gel system [52]. Delikanli et al. [53] reported that cohesiveness was related to the strength of gel composition, which reflected the water-holding properties of yogurt. In addition, specific types of EPS may be responsible for the different textures of yogurt. However, Khanal [54] reported that the reason for the different textures of yogurt is not only the concentration or molar mass of EPS. Chaudhary et al. [34] compared the effects of EPS producing and non-EPS producing strains on *Dahi*, and the results showed that among all cultures, better quality *Dahi* can be prepared by using cultures I2 and J2 (containing EPS-producing LAB). Therefore, the effect of EPS on yogurt depended on many factors. From the above results, it was found that adding *MC5* could improve the texture of yogurt, which was consistent with the rheological structure during fermentation.

### 3.4. In Vitro Antioxidant Activity (AA) of EPS in Yogurt

EPS produced by LAB has various potential health benefits and functional roles (antioxidant activity, immunomodulatory properties, anticancer, antiulcer, etc.) in human or animal health, and antioxidant activity is one of its important probiotic functions [28]. Reactive oxygen species (ROS) are natural by-products of normal aerobic metabolism or host defense mechanisms [55] that are involved in various biological processes. With increasing health awareness among consumers, natural antioxidants have received great attention from researchers because of their ability to inhibit ROS and radicals. Hydroxyl radical (OH) have a free access to cell membranes and cause tissue damage. Thus, the scavenging of these specific radicals may avoid tissue injury [56]. In general, the antioxidant mechanisms of bacterial EPS may include degradation of superoxide anion and hydrogen peroxide via ROS, inhibition of lipid peroxidation, reduction of metal ion chelation activity, and upregulation of enzymatic and non-enzymatic antioxidant activities [57].

**Figure 6:**
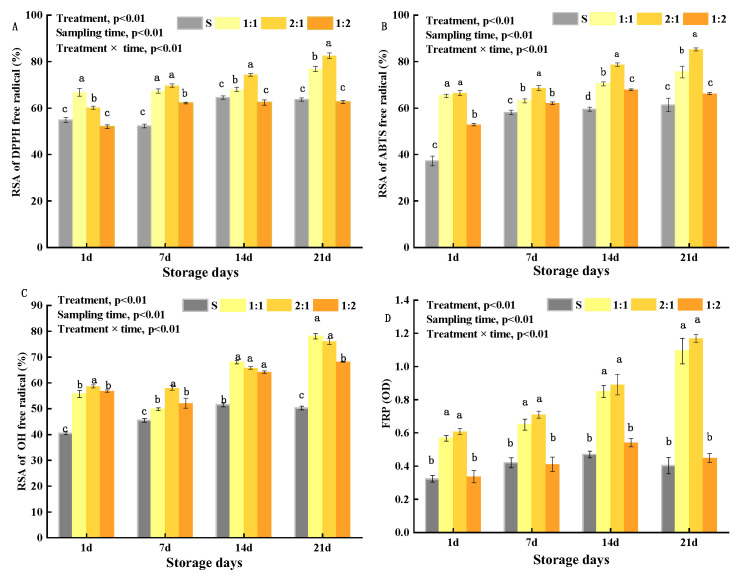
Antioxidative properties of EPS in yogurt were added with different proportions of *MC5* during storage. (**A**–**C**) Radical scavenging activity (RSA) of DPPH, ABTS, and OH, respectively. (**D**) OD of ferric iron-reducing power (FRP). Error bars represent the standard errors (se) of the model-fitted mean value (*n* = 3). “a, b, c, d” indicate significant differences within yogurt groups during storage at *p* < 0.05.

In this study, the antioxidant activity (AA) of four yogurt samples during storage was measured in terms of the RSA of DPPH, ABTS, OH, and ferric iron-reducing power (FRP). As shown in Figure 6, the antioxidant indexes (RSA of the DPPH, ABTS, OH, and FRP) of the 1:1, 2:1, and 1:2 groups were higher than those of the control S, indicating that the addition of *MC5* significantly increased the AA in yogurt samples. This may be due to increased EPS production, metabolized in yogurt samples with the prolongation of storage time, as AA was consistent with the EPS production results in 2.1. During storage of the control S, the RSA of DPPH, ABTS, and OH increased slowly, while the FRP remained constant. The RSA of DPPH, ABTS, and OH in the 1:1, 2:1, and 1:2 groups increased at higher rates during storage, especially in the 1:1 and 2:1 groups. Compared with the control S, the DPPH value, ABTS value, and OH value in the 1:1 group increased by 13.18%, 14.14%, and 27.81%, respectively, while those of the 2:1 group increased by 18.87%, 23.87%, and 25.77%, respectively, during storage. Li et al. [58] reported dose-dependent OH, DPPH, and ABTS radical scavenging activity of EPS from LAB. The contents of active metabolites (polysugars and tri-peptides) increased after 14 days of storage [20]. These explained the antioxidant activity of the yogurt samples increased during storage in this study. After 21 storage days, the RSA of DPPH, ABTS, OH, and OD_700_ of FRP of 2:1 group yogurt samples were 82.51%, 85.20%, 76.02%, and 1.17, respectively. However, the interaction between the addition ratio of *MC5* and the storage time of yogurt samples had a significant effect on AA (*p* < 0.01). These findings suggest that the *MC5* of the yogurt could donate electrons or hydrogens to scavenge free radicals.

Many studies have demonstrated the AA of EPS, with some studies reporting lower AA than the results of this study. Yang [5] reported that the RSA of the EPS solution against the DPPH and OH were 40% and 17.76%, respectively. Wang et al. [10] showed that the ABTS scavenging activity of EPS from two Porphyridium strains was 35.97% and 47.02% at 5 mg/mL concentration, and that of superoxide anion scavenging activities reached 19.38% and 7.54%, respectively. Naoki et al. [59] reported that *Lb. gasseri* MYU 1 showed the highest ORAC test value, exhibiting approximately double the activity of the control (4.03 μmol TE/g). The authors also reported ORAC values for *Lb. gasseri* MYU 1, *Lb. sakei* MYU 10, *Lb. gasseri* MYU 17, and *P. pentosaceus* MYU 759 were 11.61, 12.77, 6.16, and 4.49 μmol GAE/g, respectively in the HORAC test. The results of Zhang et al. [60] indicated that *L. plantarum* C88-EPS exhibited relatively strong RSA of OH and DPPH (85.21% and 52.23%). Therefore, the difference may be caused by the concentration of EPS and the different starters in yogurt. In addition, some scholars have found that one of the antioxidant signaling pathways is the Nrf2-controlled antioxidant response element (ARE). These antioxidant systems included superoxide dismutase, catalase, and glutathione peroxidase, and small molecules (such as albumin, ceruloplasmin, and ferritin) and macromolecules [55], which could be another reason for the difference in AA.

### 3.5. Microbiological and Physicochemical Analysis of Yogurt during Storage

In this study, the changes in LAB survival counts, pH, titratable acid (TA), and water holding capacity (WHC) of four yogurt samples were tracked and monitored to comprehensively evaluate the probiotic capability, post-acidification, and stability of *MC5* yogurt during storage. The results are shown in Table 3.

#### 3.5.1. The Number of Survival LAB in Yogurt during Storage

Table 4 shows the changes in the viable counts of *L. plantarum* *MC5*, *S. thermophilus*, and *L. delbrueckii* subsp. *bulgaricus* in the four types of yogurt samples during storage. The number of viable *S. thermophilus* and *L. delbrueckii* subsp. *bulgaricus* in four yogurt samples gradually decreased as storage time increased (Table 4). Compared with the control S, the number of *S. thermophilus* in the yogurt samples supplemented with *MC5* increased significantly (*p* < 0.05) after 14 days of storage, while the number of *L. delbrueckii* subsp. *bulgaricus* remained fairly constant. This showed that the addition of *MC5* can promote the growth of *S. thermophilus*, which may be due to the EPS and other substances produced by *MC5* in the metabolism as growth-promoting factors of S*. thermophilus*. In addition, the interaction between the addition ratio of *MC5* and the storage time of yogurt samples had a significant effect on viable counts of *MC5* and *Streptococcus thermophilus* (*p* < 0.01). Furthermore, the viable counts of *L. plantarum* *MC5* in 2:1 and 1:2 were significantly higher than that of commercial starters (*S. thermophilus* and *L. delbrueckii* subsp*. bulgaricus*) throughout the storage period (*p* < 0.05) after 21 storage days. These findings showed that *L. plantarum* *MC5* was highly stable in yogurt and met the requirements of EU and Australian standards (the number of viable LAB in probiotic products should not be less than 10^6^ CFU/g or 10^6^ CFU/mL) [61]. However, Yakult, a viable-LAB product that is now sold in many countries around the world and recognized by consumers, is fermented with *L. casei Shirota* with viable cells above 1 × 10^8^ CFU/g, which is much higher than the lowest dose that can play a role (10^6^ CFU/mL or 10^6^ CFU/g). Japan stipulated a minimum of 10^7^ CFU/g or 10^7^ CFU/mL in probiotic products. Therefore, in this study, *L. plantarum* *MC5* was relatively stable during storage, and the viable counts were above 10^8^ CFU/mL, which could fully ensure its probiotic effect.

#### 3.5.2. Analysis of pH, TA, and WHC of Four Yogurt Samples during Storage

There was no significant difference in pH and TA among all four yogurt samples on the first day of storage (*p* > 0.05); however, TA showed a significantly increasing trend as storage time progressed ((*p* > 0.05, Table 5). The fermentation time of four groups of yogurt samples was 5–6 h (pH 4.6). The pH of control S, 1:1, 2:1, and 1:2 decreased from 4.54 to 3.51, 4.55 to 4.09, 4.53 to 4.31, and 4.52 to 3.62, respectively, during storage. The results showed that the reduced speed of S was faster than those of other samples. After 14 and 21 days of storage, the pH values of the 1:1 and 2:1 yogurt samples were significantly lower than those of the control S and the 1:2 group (*p* < 0.05). This indicated that the degree of post-acidification in the 1:1 and 2:1 groups was very low and the addition of *L. plantarum* *MC5* improved the post-acidification problem yogurt samples. The TA values of 1:1, 2:1 and 1:2 yogurt samples were 97.57°T, 93.90°T, and 101.83°T after 21 storage days. The TA values of the 1:1 and 2:1 yogurt samples were significantly higher than those of the control S and the 1:2 group (*p* < 0.05). This could be due to *MC5* using the nutrients in the yogurt to produce large amounts of EPS and a small amount of lactic acid during the storage. On the contrary, the commercial starters utilized nutrients to metabolize and synthesize fewer EPS and more lactic acid and acetic acid. This could also be due to high buffering capacity of the crude EPS powder and starter culture inhibiting levels of lactic acid in yogurt [41]. In addition, the interaction between the addition ratio of *MC5* and the storage time of yogurt samples had a significant effect on pH and WHC (*p* < 0.01).

On the other hand, the WHC of control S and 2:1 yogurt samples decreased significantly with the prolongation of storage time (*p* < 0.05), while those of 1:1 and 2:1 remained constant. The WHC values of the 1:1 and 2:1 yogurt samples were significantly higher than that of the control S (*p* < 0.05) during storage, the WHC of the 1:1 and 2:1 increased by 14.56%, 23.58% than that of S, respectively, at the end of storage (Table 5). After the 21 days of storage, the WHC levels of the 1:1 and 2:1 groups (68.33% and 60.59%) were still significantly higher than those of the control S and 1:2 groups (*p* < 0.05). These results indicated that the addition of *MC5* probably improved the syneresis phenomenon of yogurt samples during storage, thereby making the stability of whey in the protein network structure higher in the 1:1 and 2:1 groups. It has been reported that milk fermented by the strain Ldb2214 producing EPS shows a good WHC value [62]. The results of Wang et al. [63] reported that the highest WHC of AE5 (adding EPS yogurt simple) was 66.23%. This implied that yogurt adding *MC5* has a stronger ability to absorb water, which can extend the shelf life of the product.

Akhtar [64] reported that denaturation of whey proteins enhanced the gelling characteristics with suitable heating and improved the surface area which allowed high WHC of yogurt, while another study, observed that EPS can bind water [65]. This water-binding ability of EPS limited the whey precipitation of yogurt and the loosening of the protein gel structure, resulting in the high cohesiveness of the yogurt samples. In addition, the water-binding ability of EPS-LAB was influenced by its type, quantity, distribution, and the interaction of protein networks with EPS, as well as the fermentation time of the yogurt. The longer fermentation time allowed for more structural rearrangements, which led to the formation of weak structures and increased spontaneous whey precipitations [66,67]. Therefore, *L. plantarum* *MC5* as a starter can greatly improve the stability of yogurt during storage.

### 3.6. Principal Component Analysis (PCA) of All Parameters

**Figure 7:**
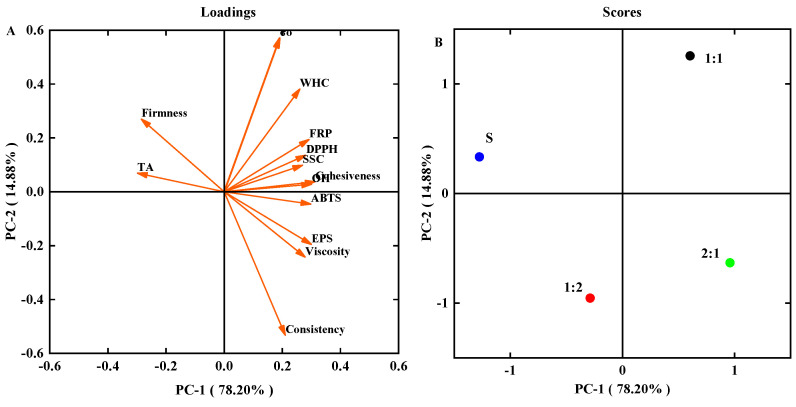
Principal component analysis (PCA) of all data. (**A**) Loading plot indicating the correlation between variables (EPS, apparent viscosity, firmness, consistency, cohesiveness, survival counts of *S. thermophilus*, DPPH, ABTS, OH, FRP, pH, TA, and WHC), EPS content, and viscosity. (**B**) Score plot indicating sample distribution based on the formulation.

A PCA was applied to evaluate correlations between the following parameters: EPS production, apparent viscosity, firmness, consistency, cohesiveness, survival counts of *S. thermophilus*, antioxidant activity (DPPH, ABTS, OH, FRP), and physical and chemical indicators (pH, TA, WHC). Figure 7A shows the obtained variable map. Principal components 1 and 2 accounted for 78.20% and 14.88% of the total variance, respectively. A high correlation (R = 0.976) between EPS content and apparent viscosity of yogurt samples was observed, indicating that this parameter can be useful to monitor the quality change of the yogurt samples. Firmness and TA were negatively correlated with EPS content, apparent viscosity, and consistency. To better discriminate the four yogurt samples, a scores plot was performed according to PC1 and PC2 (Figure 7B). Yogurt samples supplemented with *MC5* were separated from the control S, which showed that adding an *MC5* starter affected the quality of yogurt. Furthermore, 2:1 and 1:1 samples were located on the positive sides of PC1, whereas 1:2 and control S were scattered along the negative sides of PC1. This supported the earlier findings made in this study, the yogurt properties of the 2:1 and 1:1 samples were better than 2:1 and control S.

## 4. Conclusions

The results showed that the addition of *L. plantarum* *MC5* can stimulate metabolic activities of starters making them produce a large number of EPS content, which can be applied in the yogurt-making process. A more favorable apparent viscosity, modulus of elasticity, and antioxidant activity were obtained in the formulations using *L. plantarum MC5* during storage. At the same time, the yogurt samples fermented with *L. plantarum* *MC5* had the highest WHC and greatly improved syneresis during storage, indicating that adding *MC5* could improve the stability of yogurt samples during storage. Notably, the addition of *MC5* also had a growth-promoting effect on *S. thermophilus* in yogurt samples during storage. Therefore, the above results of this study revealed that the quality of yogurt produced from the combination of *MC5* and commercial starters was better than that of control yogurt. Moreover, the optimal yogurt properties were obtained by 2:1 sample formulation, followed by 1:1 sample.

In a nutshell, the study highlighted the potential of *MC5* strain as a compound starter and its contribution to improving the rheological, texture features, and storage stability of yogurt. The yogurt containing probiotic *MC5* met consumers’ demands for naturalness and health. However, further research is needed on the metabolic mechanism of EPS under the interaction effect between LAB, and the structural properties of this EPS.

## Figures and Tables

**Table 1:** Composition of Holstein milk.

Composition of Cow Milk	Fat	SNF	Protein	Lactose	Ash Content	Total Solids
Percentage (%)	3.28	10.01	3.82	5.43	0.73	13.29

Notes: one batch of milk was measured three times, and the results were average (*n* = 3).

**Table 2:** Inoculated amount of *MC5* and starters in four groups of yogurt samples.

Types of Yogurt	*L. plantarum* *MC5* (%)	Starters S (%)
Control group S	-	3
*MC5*:S = 1:1	1.5	1.5
*MC5*:S = 2:1	2	1
*MC5*:S = 1:2	1	2

Notes: the experiment was repeated in triplicates.

**Table 3:** Rheological parameters were obtained using the Herschel–Bulkley model for yogurt samples during storage at 4 °C. “a, b, c, d” indicate significant differences within yogurt groups during storage at *p* < 0.05.

Group	Storage Time (d)	σ_o (Pa)_	K (Pa.s^n^)	*n*	R^2^
1:1	1d	3.26 ± 0.02 ^d^	2.51 ± 0.04 ^d^	0.18	0.9999
	7d	11.20 ± 0.09 ^c^	7.64 ± 0.01 ^c^	0.23	0.9993
	14d	46.70 ± 0.27 ^b^	16.2 ± 0.04 ^b^	0.18	0.9997
	21d	66.90 ± 1.19 ^a^	18.79 ± 0.03 ^a^	0.60	0.9929
2:1	1d	16.56 ± 0.14 ^d^	4.70 ± 0.02 ^c^	0.05	0.9983
	7d	25.99 ± 0.18 ^c^	3.17 ± 0.03 ^d^	0.17	0.9966
	14d	34.01 ± 0.13 ^b^	21.1 ± 0.06 ^b^	0.17	0.9998
	21d	54.79 ± 0.58 ^a^	49.09 ± 0.16 ^a^	0.28	0.9963
1:2	1d	17.73 ± 0.17 ^d^	5.00 ± 0.02 ^d^	0.59	0.9958
	7d	24.39 ± 0.19 ^c^	6.36 ± 0.04 ^c^	0.22	0.9962
	14d	27.75 ± 0.11 ^b^	12.93 ± 0.03 ^b^	0.01	0.9982
	21d	46.01 ± 0.27 ^a^	19.34 ± 0.02 ^a^	0.47	0.9978
S	1d	3.15 ± 0.02 ^d^	2.08 ± 0.02 ^d^	0.67	0.9956
	7d	10.28 ± 0.06 ^c^	3.22 ± 0.03 ^c^	0.18	0.9992
	14d	23.87 ± 0.09 ^b^	10.67 ± 0.06 ^b^	0.89	0.9903
	21d	49.41 ± 1.25 ^a^	54.45 ± 0.21 ^a^	0.05	0.9918
Treatment	*p*	<0.01	<0.01	<0.01	-
Time		<0.01	<0.01	<0.01	-
Treatment × Time		<0.01	<0.01	0.07	-

**Table 4:** Quantities of commercial starters and MC5 survival counts (10^8^ CFU/mL) in four groups of yogurt during storage. Data represented as the model-fitted mean ± standard errors. “A, B, C, D” indicate significant differences within yogurt groups in different storage at *p* < 0.05. “a, b, c, d” indicate significant differences within different strains of the same storage time at *p* < 0.05.

The Type of LAB	Time (d)	Control S	1:1	2:1	1:2	Treatment	Time	Treatment × Time
*L. plantarum MC5*	1	-	3.95 ± 0.07 ^Aa^	3.80 ± 0.09 ^Aa^	3.69 ± 0.08 ^Aa^	*p*	*p*	*p*
	7	-	3.61 ± 0.09 ^Bb^	3.68 ± 0.09 ^Ab^	3.46 ± 0.08 ^Bb^	<0.01	<0.01	<0.01
	14	-	3.24 ±0.09 ^Ca^	3.17 ± 0.09 ^Ba^	3.19 ± 0.09 ^Ca^			
	21	-	3.12 ±0.09 ^Ca^	2.75 ± 0.09 ^Cb^	2.96 ± 0.08 ^Da^			
*S. thermophilus*	1	3.70 ± 0.07 ^Aa^	3.89 ± 0.08 ^Aa^	3.95 ± 0.07 ^Aa^	3.54 ± 0.02 ^Ab^	*p*		*p*
	7	3.44 ± 0.03 ^Bb^	3.53 ± 0.04 ^Bb^	3.82 ± 0.03 ^Ba^	3.40 ± 0.01 ^Bb^	<0.01	<0.01	<0.01
	14	2.98 ± 0.07^Cb^	3.15 ± 0.09 ^Ca^	3.34 ± 0.09 ^Ca^	3.10 ± 0.02 ^Cb^			
	21	1.10 ± 0.03 ^Dd^	3.16 ± 0.08 ^Ca^	2.55 ± 0.08 ^Db^	2.47 ± 0.04 ^Db^			
*L. delbrueckii* subsp. *bulgaricus*	1	3.46 ± 0.06 ^Ab^	3.28 ± 0.07 ^Ac^	3.53 ± 0.04 ^Ab^	3.25 ± 0.09 ^Ac^	*p*		*p*
	7	3.01 ± 0.05 ^Bc^	2.89 ± 0.03 ^Bd^	3.09 ± 0.08 ^Bc^	2.72 ± 0.04 ^Bd^	<0.01	<0.01	0.63
	14	2.96 ± 0.05 ^Bb^	2.84 ± 0.03 ^Bc^	2.94 ± 0.04 ^Bd^	2.72 ± 0.08 ^Bd^			
	21	1.02 ± 0.02 ^Cd^	1.11 ± 0.02 ^Cd^	1.58 ± 0.08 ^Dc^	1.70 ± 0.07 ^Cc^			

**Table 5:** Analysis of pH, TA, and WHC of four yogurt samples during storage. Data represented as the model-fitted mean ± standard errors. “a, b, c, d, e” indicate significant differences within yogurt groups during storage at *p* < 0.05.

Index	Time (d)	Control S	1:1	2:1	1:2	Treatment	Time	Treatment × Time
pH	1	4.54 ± 0.06 ^a^	4.55 ± 0.06 ^a^	4.53 ±0.02 ^a^	4.52 ±0.02 ^a^	*p*	*p*	*p*
	7	4.27 ± 0.01 ^b^	4.22 ± 0.03 ^b^	4.50 ± 0.01 ^a^	4.49 ± 0.02 ^a^	<0.01	<0.01	<0.01
	14	3.84 ± 0.06 ^c^	4.13 ± 0.06 ^b^	4.41 ± 0.02 ^a^	3.96 ± 0.06 ^c^			
	21	3.51 ± 0.03 ^d^	4.09 ± 0.07 ^b^	4.31 ± 0.06 ^b^	3.62 ± 0.06 ^d^			
	range	1.03	0.46	0.22	0.90			
TA (°T)	1	74.57 ± 0.88 ^a^	75.00 ± 1.02 ^a^	72.17 ± 0.72 ^a^	74.46 ± 0.76 ^a^	*p*	*p*	*p*
	7	90.33 ± 1.53 ^c^	84.97 ± 1.55 ^b^	85.27 ± 0.73 ^b^	91.53 ± 0.95 ^c^	<0.01	<0.01	0.072
	14	99.50 ± 1.05 ^de^	95.07 ± 0.57 ^cd^	92.43 ± 1.27 ^c^	98.80 ± 0.58 ^d^			
	21	103.28 ± 1.70 ^e^	97.57 ± 0.68 ^d^	93.90 ± 1.80 ^c^	101.83 ± 1.01 ^e^			
	range	28.71	22.57	21.73	27.37			
WHC (%)	1	71.22 ± 1.71 ^b^	82.36 ± 0.68 ^a^	84.17 ± 2.41 ^a^	81.62 ± 2.26 ^a^	*p*	*p*	*p*
	7	63.38 ± 1.79 ^c^	71.18 ± 1.99 ^b^	82.65 ± 1.79 ^a^	74.52 ± 1.89 ^ab^	<0.01	<0.01	<0.01
	14	57.19 ± 0.34 ^d^	70.22 ± 1.84 ^b^	75.65 ± 1.81 ^a^	66.24 ± 0.95 ^c^			
	21	50.61 ± 0.72 ^e^	67.80 ± 1.73 ^b^	60.59 ± 0.93 ^c^	53.74 ± 1.08 ^e^			
	range	20.61	14.56	23.58	27.88			

## Data Availability

The data presented in this study are available upon request from the corresponding author. The data are not publicly available due to privacy.

## Abbreviations

EPS	Exopolysaccharide
LAB	Lactic acid bacteria
AA	Antioxidant activity
RSA	Radical scavenging activity
DPPH	1,1-Diphenyl-2-picrylhydrazyl, (free radical)
ABTS	2,2’-Azinobis (3-ethylbenzothiazoline-6-sulfonic acid ammonium salt)
OH	Hydroxyl (free radicals)
FRP	Ferric reducing power
TA	Titratable acidity
WHC	Water holding capacity
PCA	Principal component analysis
